# Analysis of Time Between Skin Lesion and Lymph Node Biopsies and Lymph Node Metastasis in Patients With Melanoma

**DOI:** 10.1001/jamanetworkopen.2023.11472

**Published:** 2023-05-03

**Authors:** Elliot L. H. Le, Emma Lamping, Laura Helmkamp, Janice Bone, Martin McCarter, Nicole Kounalakis, Camille Stewart

**Affiliations:** 1 Division of Surgical Oncology, University of Colorado School of Medicine, Anschutz Medical Campus, Aurora; 2 Adult and Child Center for Outcomes Research & Delivery Science, University of Colorado School of Medicine, Aurora; 3 Melanoma & Sarcoma Specialists of Georgia, Northside Hospital Cancer Institute, Atlanta; 4 Division of Plastic and Reconstructive Surgery, University of Colorado School of Medicine, Anschutz Medical Campus, Aurora

## Abstract

This cohort study assesses whether increasing time to surgery is associated with sentinel lymph node status in patients with cutaneous melanoma stage T1b or higher.

## Introduction

The standard of care treatment for patients with stage T1b or higher cutaneous melanoma without radiographic or clinical evidence of metastatic disease is wide local excision (WLE) and sentinel lymph node biopsy (SLNB).^1^ The immediate purposes of WLE and SLNB are prognostication, staging via identification of occult metastatic disease, and guiding decisions for adjuvant systemic treatment.^2,3^ It is unclear, however, how long a patient can safely wait between diagnosis and surgery before it can affect disease staging. We sought to determine whether increasing time to surgery was associated with sentinel lymph node status.

## Methods

This cohort study followed the Strengthening the Reporting of Observational Studies in Epidemiology (STROBE) reporting guidelines. The University of Colorado Hospital and Northside Hospital institutional review boards approved this study with a waiver of informed consent because the study used deidentified data, in accordance with 45 CFR § 46. The prospectively maintained melanoma databases of University of Colorado Hospital (academic hospital) and Northside Hospital (community hospital) were queried for patients with primary T1b to T4, clinical N0M0 cutaneous melanoma who underwent WLE and SLNB from January 2018 to June 2021. The primary outcome was sentinel lymph node status as a function of time from diagnosis to surgery. Race and ethnicity (African American or Black, Asian, Pacific Islander, and White races and Hispanic ethnicity) information was derived from the electronic health record and was patient reported; race and ethnicity were analyzed in this study to ensure the data reflect the population at large. Multivariable logistic regression with a significance threshold of *P*  <  .05 was used to determine odds of node positivity with time to surgery adjusted for covariates of interest. Locally estimated scatterplot smoothing was used to visualize the association between time to surgical treatment and node positivity and to search for possible nonlinearities using SAS statistical software, version 9.4, (SAS Institute). Data analysis was performed from October 2021 to August 2022.

## Results

A total of 642 patients (median [IQR] age, 60.0 [49.0-70.0] years; 367 men [57.2%]; 624 White patients [97.2%]) with melanoma underwent WLE and SLNB within 90 days of diagnosis; 412 patients (64.3%) had T1 or T2 stage disease at diagnosis. A total of389 patients (60.6%) underwent surgery at the community hospital (Table). Of the patients who underwent surgery, 253 (39.4%) underwent surgery within 30 days of diagnosis, 323 (50.3%) underwent surgery within 31 to 60 days of diagnosis, and 66 (10.3%) underwent surgery within 61 to 90 days of diagnosis. The nodal positivity rate was 20.2% (130 of 642 patients). Nodal positivity rates for patients who underwent surgery within 30 days of diagnosis were not significantly different from those of patients who underwent surgery within 31 to 60 days of diagnosis (odds ratio [OR], 0.84; 95% CI, 0.54-1.29) or 61 to 90 days of diagnosis (OR, 0.80; 95% CI, 0.40-1.61) when adjusting for treatment location alone. Multivariable logistic regression adjusting for treatment location, age, sex, race, T stage, ulceration, and mitoses also did not find a statistically significant association of a final N stage greater than 0 with a time to surgery of 31 to 60 days (OR, 0.79; 95% CI, 0.50-1.23) or 61 to 90 days (OR, 0.77; 95% CI, 0.37-1.63). Younger age (OR for 10-year increase in age, 0.76; 95% CI, 0.65-0.88) and higher T stage (OR for T4, 14.57; 95% CI, 5.97-35.58) were significantly associated with a final N stage greater than 0. When repeat analyses were limited to patients with stage T3 to T4 disease and when time to surgery was analyzed as a continuous variable, time to surgery was not associated with a final N stage greater than 0 (Figure). Analysis before and after the COVID-19 pandemic did not show significant increases in time to surgery.

**Table.** Demographic Characteristics of Patients With Primary T1b to T4, Clinical N0M0 Cutaneous Melanoma Who Underwent Wide Local Excision and Sentinel Lymph Node Biopsy Within 90 Days of Diagnosis

Characteristic	Patients, No. (%) (N = 642)	Positive nodes, No. (%)	OR (95% CI)	
			Multivariable regression adjusted for site effect only	Multivariable regression adjusted for all covariates
Location				
Northside Hospital	389 (60.6)	62 (15.9)	1 [Reference]	1 [Reference]
University of Colorado Hospital	253 (39.4)	68 (26.9)	1.93 (1.31-2.86)^a^	2.15 (1.37-3.37)^a^
Age, median (IQR), y	60.0 (49.0-70.0)	NA	0.85 (0.76-0.99)^a^	0.76 (0.65-0.88)^a^
Sex				
Female	275 (42.8)	54 (19.6)	0.92 (0.62-1.36)	0.89 (0.57-1.37)
Male	367 (57.2)	76 (20.7)	1 [Reference]	1 [Reference]
Race^b^				
White	624 (97.2)	126 (20.2)	1 [Reference]	1 [Reference]
Other or unknown^c^	18 (2.8)	4 (22.2)	1.01 (0.32-3.17)	0.76 (0.21-2.72)
T stage				
T1	180 (28.1)	10 (5.6)	1 [Reference]	1 [Reference]
T2	232 (36.2)	41 (17.71)	3.94 (1.90-8.15)^a^	3.55 (1.69-7.48)^a^
T3	162 (25.3)	48 (29.6)	7.36 (3.56-15.22)^a^	6.72 (3.12-14.45)^a^
T4	67 (10.5)	31 (46.3)	16.15 (7.18-36.29)^a^	14.57 (5.97-35.58)^a^
Ulcerated				
No	434 (67.8)	65 (15.0)	1 [Reference]	1 [Reference]
Yes	206 (32.2)	65 (31.6)	2.50 (1.68-3.72)^a^	1.44 (0.89-2.33)
Mitosis				
Absent	49 (7.6)	3 (6.1)	0.21 (0.06-0.68)^a^	0.47 (0.13-1.68)
Present	573 (89.3)	126 (22.0)	1 [Reference]	1 [Reference]
Unknown	20 (3.1)	1 (5.0)	0.17 (0.02-1.32)	0.32 (0.04-2.57)
Final N stage				
N0	512 (79.8)	NA	NA	NA
Greater than N0	130 (20.2)	NA	NA	NA
Time to treatment, d				
0-30	253 (39.4)	50 (19.8)	1 [Reference]	1 [Reference]
31-60	323 (50.3)	66 (20.4)	0.84 (0.54-1.29)	0.79 (0.50-1.23)
61-90	66 (10.3)	14 (21.2)	0.80 (0.40-1.61)	0.77 (0.37-1.63)

Abbreviations: NA, not applicable; OR, odds ratio.

^a^ Denotes statistical significance.

^b^ Race was obtained from the electronic health record and is patient reported.

^c^ Other includes African American or Black, Asian, and Pacific Islander races and Hispanic ethnicity.

**Figure.**
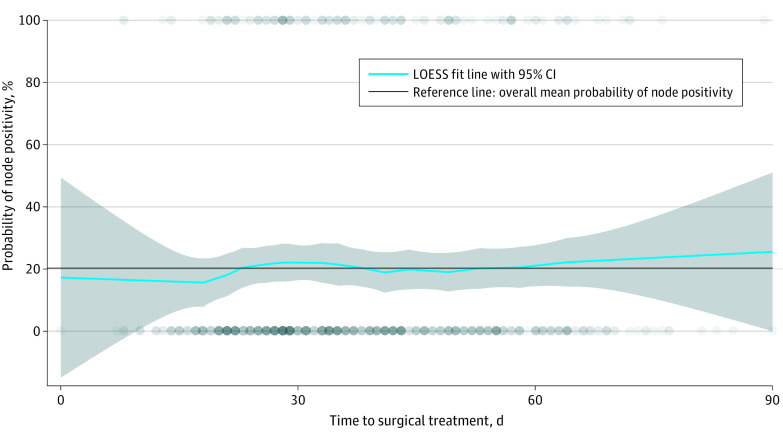
Locally Estimated Scatterplot Smoothing (LOESS) of Sentinel Lymph Node Positivity Graph shows smoothed curve LOESS of sentinel lymph node positivity compared with time from diagnosis to surgical treatment for patients with primary stage T1b to T4, clinical N0M0 cutaneous melanoma. This curve shows near constant probability of node positivity by time to surgical treatment in this sample up to 90 days after diagnosis. The reference line of 20.2% gives the overall mean rate of node positivity in this sample. Shaded area denotes 95% CIs, and circles denote individual patient data points, with darker circles representing overlapping data points.

## Discussion

This cohort study indicated that odds of sentinel lymph node positivity remained similar over time up to 90 days for those undergoing WLE and SLNB for T1b or higher clinical N0M0 cutaneous melanoma when using multivariable regression accounting for treatment location, age, sex, race, T stage, ulceration, and mitoses. These results suggest that, although surgery for melanoma should be offered in a timely manner, time is not associated with a change in the risk of upstaging disease within 90 days of diagnosis, potentially alleviating clinician and patient anxiety when a delay within this time frame is unavoidable (eg, operating room or staffing availability) or may be desired by the patient (eg, upcoming major life event). Previous studies^4,5,6^ have reported negative associations between time to surgery and melanoma survival, but did not examine how time was associated with the identification of occult metastatic disease and were conducted before the widespread use of effective systemic therapies for melanoma, thereby limiting their application to current clinical practice. Limitations of this study are its retrospective nature and number of treatment sites. Additional larger studies corroborating these findings are warranted.

## Notes

Supplement.
Data Sharing Statement
