# Supplementary material for: Analysis of Time Between Skin Lesion and Lymph Node Biopsies and Lymph Node Metastasis in Patients With Melanoma
Source: JAMA Netw Open. 2023 May 3;6(5):e2311472. doi: 10.1001/jamanetworkopen.2023.11472 (PMC10157428; doi:10.1001/jamanetworkopen.2023.11472)
Supplement: Supplement. — Data Sharing Statement [file jamanetwopen-e2311472-s001.pdf]

Data Sharing Statement

Le. Analysis of Time Between Skin Lesion and Lymph Node Biopsies and Lymph Node Metastasis in Patients With Melanoma. *JAMA Netw Open*. Published May 03, 2023. doi:10.1001/jamanetworkopen.2023.11472

Data
Data available: No
